# Supplementary figures and images for: Longitudinal Study of the Effects of Flammulina velutipes Stipe Wastes on the Cecal Microbiota of Laying Hens
Source: mSystems. 2022 Dec 13;8(1):e00835-22. doi: 10.1128/msystems.00835-22 (PMC9948703; doi:10.1128/msystems.00835-22)

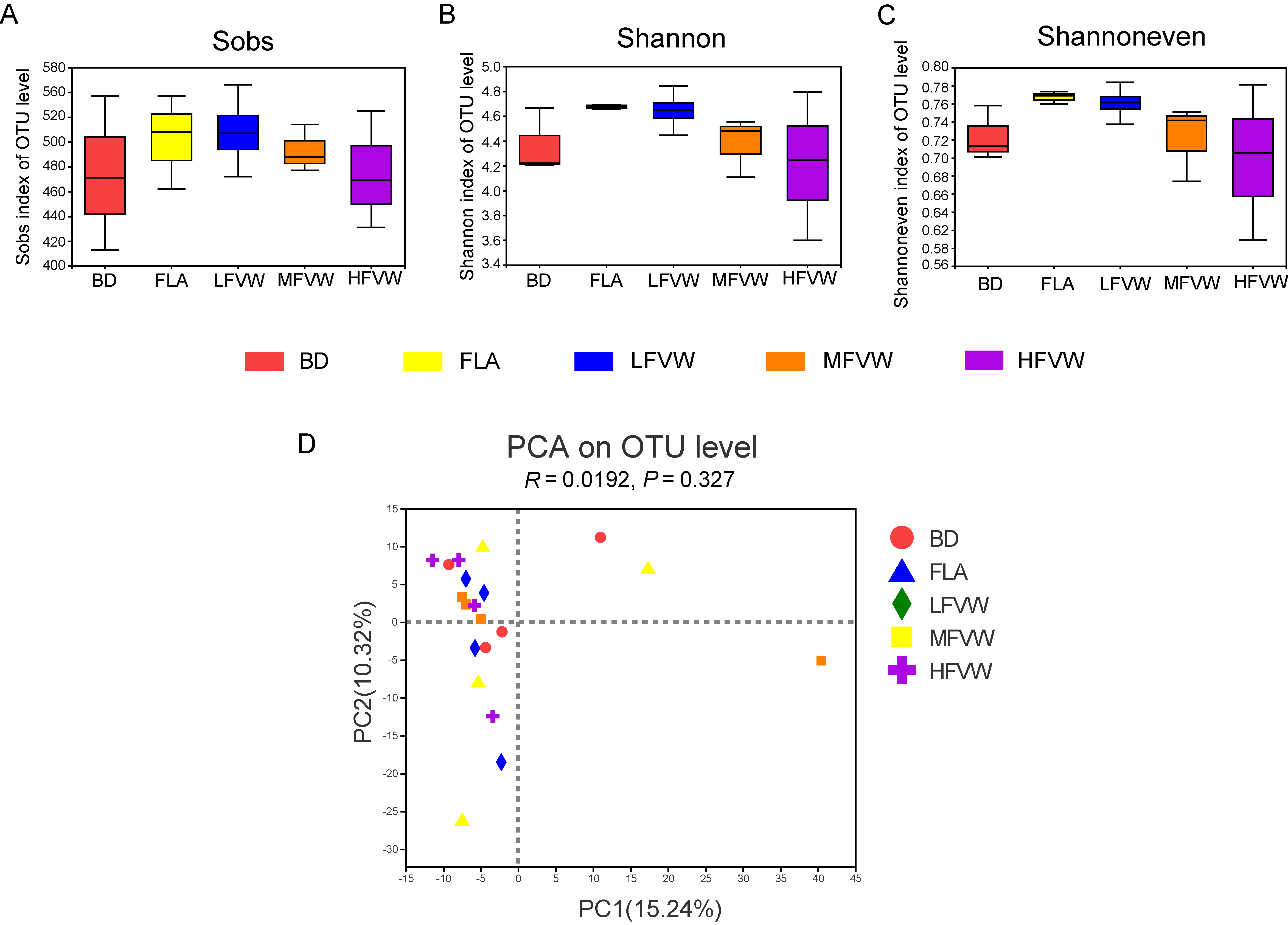

Supplement: FIG S1 [file msystems.00835-22-s0001.tif]

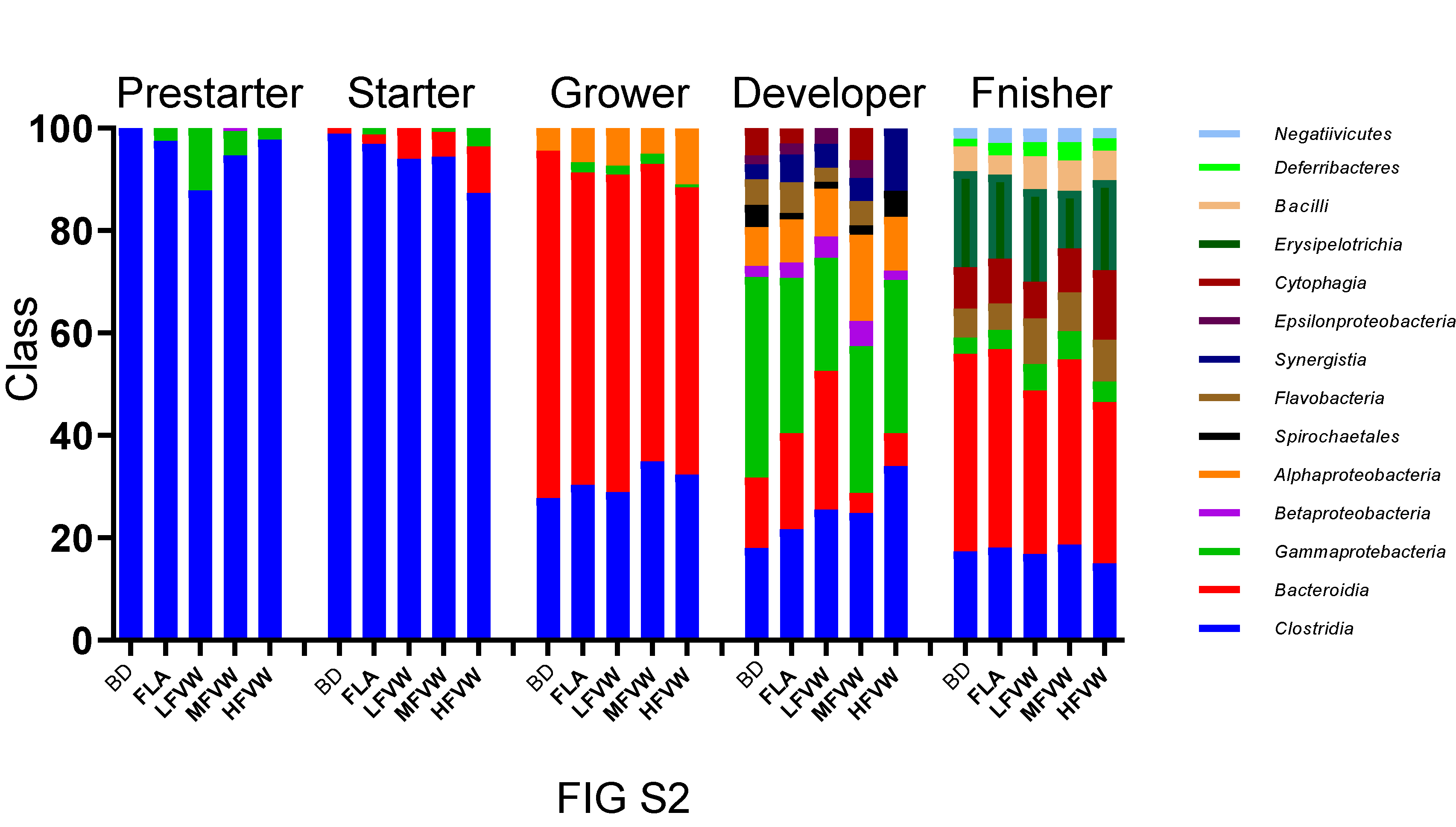

Supplement: FIG S2 [file msystems.00835-22-s0002.tif]

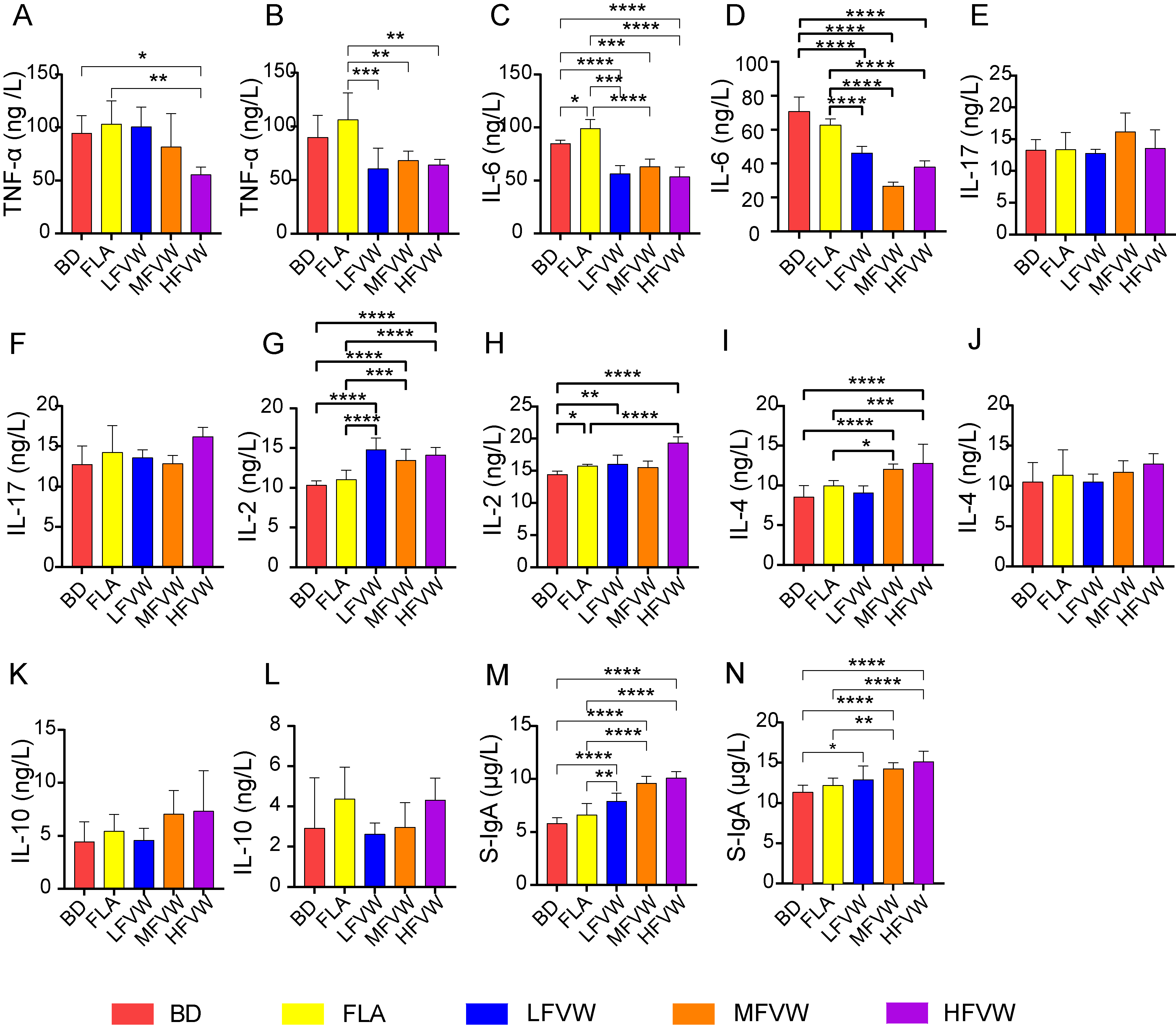

Supplement: FIG S3 [file msystems.00835-22-s0003.tif]
