# Supplementary material for: Longitudinal Study of the Effects of Flammulina velutipes Stipe Wastes on the Cecal Microbiota of Laying Hens
Source: mSystems. 2022 Dec 13;8(1):e00835-22. doi: 10.1128/msystems.00835-22 (PMC9948703; doi:10.1128/msystems.00835-22)
Supplement: TABLE S1 [file msystems.00835-22-s0004.docx]

**TABLE S1** The nutrients of *Flammulina velutipes* stipe wastes (air dry basis, %)

| **Item** | **Content** |
| --- | --- |
| Moisture | 6.41 ± 0.51 |
| Organic matter (OM) | 93.59 ± 0.51 |
| Ether extract (EE) | 9.45 ± 0.71 |
| Crude fiber (CF) | 12.38 ± 0.14 |
| Crude protein (CP) | 13.33 ± 0.04 |
| Crude ash (CA) | 7.17 ± 0.11 |
| Neutral detergent fiber (NDF) | 46.57 ± 0.42 |
| Acid detergent fiber (ADF) | 22.33 ± 0.67 |
| Soluble dietary fiber (SDF) | 5.05 ± 0.07 |
| Insoluble dietary fiber（IDF） | 39.58 ± 0.48 |
| Total dietary fiber (TDF) | 44.63 ± 0.54 |
| Hemicellulose（HCEL） | 25.37 ± 0.67 |
| Cellulose（CEL） | 18.64 ± 0.13 |
| Lignin | 3.66 ± 0.07 |
| Crude polysaccharide | 6.17 ± 0.11 |
| Calcium (Ca) | 0.89 ± 0.14 |
| Total phosphorus (P) | 0.74 ± 0.08 |

^a^Values are expressed as mean ± standard deviation (n=3).
