# Supplementary material for: Longitudinal Study of the Effects of Flammulina velutipes Stipe Wastes on the Cecal Microbiota of Laying Hens
Source: mSystems. 2022 Dec 13;8(1):e00835-22. doi: 10.1128/msystems.00835-22 (PMC9948703; doi:10.1128/msystems.00835-22)
Supplement: TABLE S5 [file msystems.00835-22-s0008.docx]

**TABLE S5** Finisher phase (113-490 days) composition and nutrient levels of diets (air-dry basis, %)

| **Items** | **BD** | **FLA** | **LFVW** | **MFVW** | **HFVW** |
| --- | --- | --- | --- | --- | --- |
| Maize corn | 55.70 | 55.65 | 54.40 | 52.50 | 51.00 |
| Soybean meal | 28.20 | 28.20 | 27.70 | 27.40 | 27.20 |
| Soya oil | 2.80 | 2.80 | 2.60 | 2.80 | 2.50 |
| FVW^1^ | - | - | 2.00 | 4.00 | 6.00 |
| Lysine | 0.20 | 0.20 | 0.20 | 0.20 | 0.20 |
| Methionine | 0.25 | 0.25 | 0.25 | 0.25 | 0.25 |
| Dicalcium | 3.60 | 3.60 | 3.60 | 2.60 | 3.60 |
| Limestone | 8.80 | 8.80 | 8.80 | 8.80 | 8.80 |
| Salt | 0.25 | 0.25 | 0.25 | 0.25 | 0.25 |
| Vit – mineral ^a^ | 0.20 | 0.20 | 0.20 | 0.20 | 0.20 |
| Antibiotic | - | 0.05 | - | - | - |
| Total | 100.00 | 100.00 | 100.00 | 100.00 | 100.00 |
| Chemical analysis ^b^ | | | | | |
| CP | 17.00 | 17.00 | 16.96 | 16.96 | 17.03 |
| Ca | 4.11 | 4.11 | 4.11 | 4.11 | 4.11 |
| P | 0.72 | 0.72 | 0.72 | 0.72 | 0.72 |
| EE | 5.23 | 5.23 | 5.22 | 5.04 | 4.93 |
| CF | 2.56 | 2.56 | 2.96 | 3.37 | 3.79 |
| Calculated analysis | | | | | |
| ME（MJ/kg） | 11.70 | 11.69 | 11.70 | 11.71 | 11.71 |
| Lysine | 1.05 | 1.05 | 1.05 | 1.05 | 1.05 |
| Methionine | 0.50 | 0.50 | 0.50 | 0.50 | 0.50 |
| Cystine | 0.28 | 0.28 | 0.28 | 0.28 | 0.28 |

^a^ Premix contained the following compound per kg: VA 4500 IU , VD 31200 IU , DL-α-tocopheryl acetate 2500 IU, VB1 5000mg, VB2 20000mg, VK 10000mg, Niacin 45000 mg, pantothenic acid 35000 mg, biotin 1500 mg, folic acid 3000 mg, vitamin B12 40 mg, zinc 45 mg, manganese 50 mg, iron 30 mg, copper 4 mg, cobalt 100 μg iodine 1 mg, selenium 100 μg.

^b^ DM=dry matter; ME= metabolizable energy; CP=crude; Ca=calcium; P=phosphorus; EE=ether extract; CF=crude fiber.
